# Bridging the gap to therapeutic strategies based on connexin/pannexin biology

**DOI:** 10.1186/s12967-016-1089-0

**Published:** 2016-11-29

**Authors:** Christian C. Naus, Christian Giaume

**Affiliations:** 1 Department of Cellular and Physiological Sciences, Faculty of Medicine, Life Sciences Institute, University of British Columbia, 2350 Health Sciences Mall, Vancouver, BC V6T 1Z3 Canada; 2 ICIRB, CNRS UMR7241/INSERM U1050, Collège de France, Paris Cedex 05, France

**Keywords:** Gap junctions, Connexins, Pannexins, Therapeutics, Peptides, Strategies

## Abstract

A unique workshop was recently held focusing on enhancing collaborations leading to identify and update the development of therapeutic strategies targeting connexin/pannexin large pore channels. Basic scientists exploring the functions of these channels in various pathologies gathered together with leading pharma companies which are targeting gap junction proteins for specific therapeutic applications. This highlights how paths of discovery research can converge with therapeutic strategies in innovative ways to enhance target identification and validation.

## Background

**Fig. 1:**
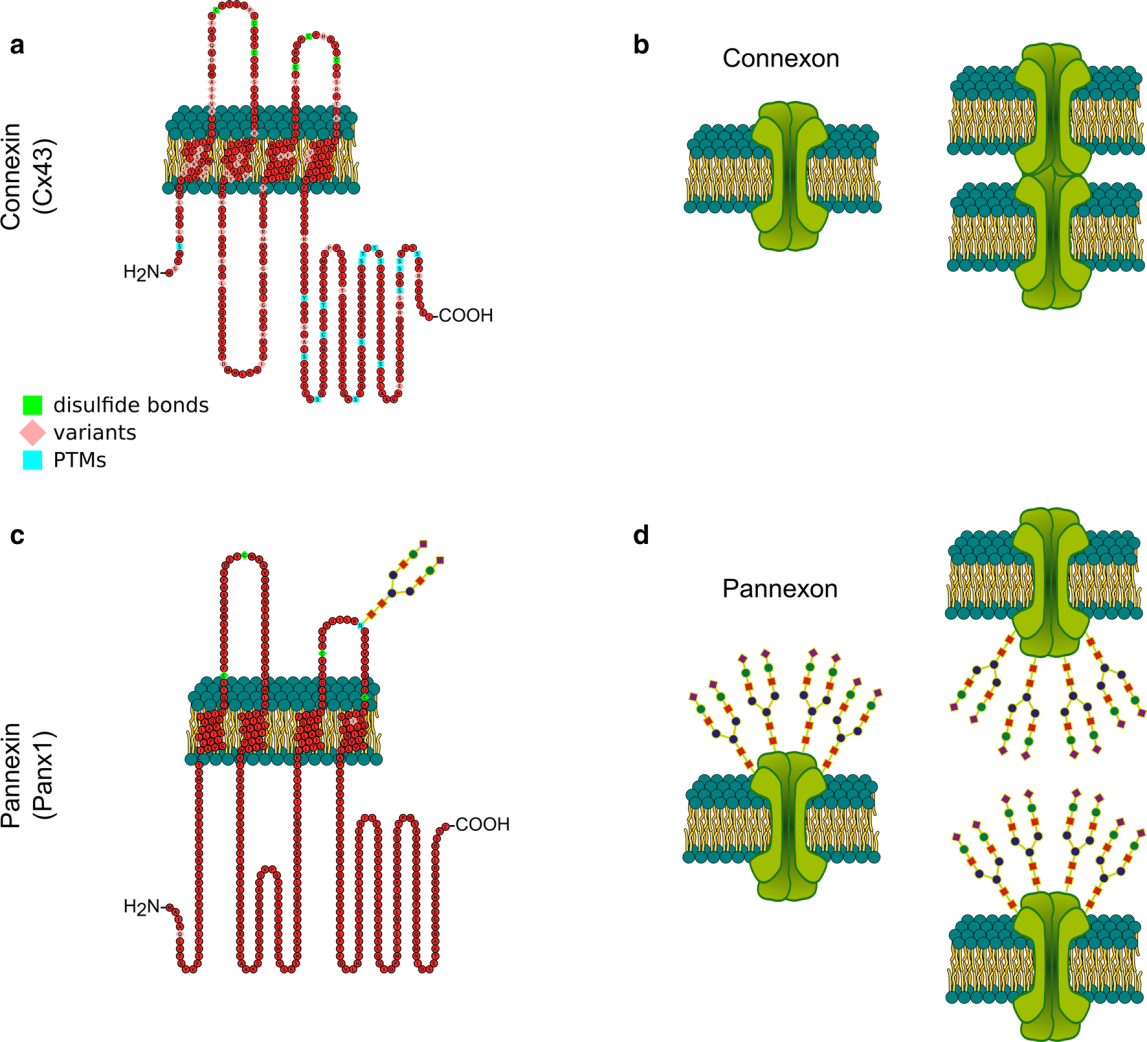
Connexin and pannexin topology. Connexin and pannexin share a common structural topology despite the absence of sequence homology. Both are transmembrane proteins with four transmembrane domains, two extracellular loops, one cytoplasmic loop, and cytoplasmic N– and C–terminal domains (**a**, **c**). Connexin and pannexin monomers both oligomerize to form a functional ‘connexon’ and ‘pannexon’ channel, respectively. However, only connexin channels can assemble into a gap junction that allows intercellular communication. Pannexin is glycosylated and large glycan residues prevent the docking of pannexin channels from adjacent cells (**b**, **d**). The amino acid sequences in **a** and **c** represent human Cx43 and Panx1 respectively. *PTMs* post-translational modifications. Figure contributed by Dr. M. Le Vasseur, University of British Columbia, Canada

The essential role of gap junction channels and hemichannels in the normal functioning of cells and its relationship to diseases is only beginning to be understood. Gap junction channels assemble when connexins oligomerize into a connexon or hemichannel and dock with another from a neighboring cell [1]. These channels cluster at defined cell–cell contacts to form gap junctions, but they can also function as single membrane hemichannels, particularly in pathological situations [2] (Fig. 1). It is now apparent that most human cells express more than one of the 21 members of the connexin family. The complexity of channel constituents and arrangements within tissues is thought to be critical in selective passage of small biological molecules, like second messengers, amino acids and metabolites. Alternatively, hemichannels allow direct communication between the cytosol and the extracellular space. Interestingly, mutations in the genes encoding several members of the connexin family have been linked to a number of human diseases [3]. In addition, the generation of connexin knockout (KO) mice has revealed a number of defects ranging from embryonic lethal to relatively normal animals [4]. The more recent discovery of pannexins, which share sequence homology to the invertebrate gap junction proteins innexins, has broadened the topic significantly [5] (Fig. 1). Pannexins form large single membrane channels serving a role in paracrine signaling that can be detrimental and contribute to cell death. These findings highlight the importance of connexins/pannexins in nearly every major organ in the body. Therapeutic interest arising from the biological insights of these proteins is beginning to gain momentum and open the way to new therapeutic interventions. Therefore a unique and timely “*Workshop on the interface between connexin/pannexin biology and therapeutics*” was held in Paris March 3–4, 2016 for the research community to interface with Biotech and Pharma companies to accelerate the innovation and application of therapeutics for a variety of diseases in which these channels are involved.

### Regulation of expression and function

When considering a therapeutic biological target such as a membrane channel, basic aspects concerning expression, regulation and function are foundational. Following an overview presented by Dale Laird (Western University, Ontario, Canada), Paul Lampe (Fred Hutchinson Cancer Research Centre, Seattle, WA, USA) provided insight on the most widely expressed gap junction protein, connexin43 (Cx43), particularly focusing on its phosphorylation at many amino acid residues in the cytoplasmic, C-terminal region [6]. These phosphorylation events, which control assembly, gating and turnover of connexin channels, are mediated via a “kinase program” where sequential phosphorylations take place and affect Cx43 channel functions. Interestingly, the complex regulation of channel assembly and turnover provides several opportunities for potential drug intervention. Dale Laird explained how cell cultures and genetically-modified mouse models are used to investigate the genotype/phenotype relationships in Cx43-linked diseases [3]. Using tissue-relevant cells derived from these mice along with patient cells, he determined the breadth of mechanisms linking Cx43 mutations to disease. For one such human disease, oculodentodigital dysplasia (ODDD), he has used the innovative approach of generating human induced pluripotent stem cells from patients and familial controls to examine the role of Cx43 in the skeleton. He stressed that a multipronged approach is necessary to investigate the molecular basis of Cx43-linked disease and that this approach allows identification of sub-classifications of disease-causing mutations necessary for personalized therapeutic intervention.

### At the heart of vascular diseases

Gap junctions sustain the regular heart beat. Thus it is no surprise to see innovations targeting gap junction channels with regard to cardiac disease. However, Mario Delmar (New York University, USA) has focused his work on channel-independent functions of Cx43. He has identified Cx43 in the heart as part of a molecular complex that includes molecules of the desmosome and of the sodium channel complex [7]. He proposed that Cx43 is part of a protein interacting network, “the connexome”, where all the proteins interacting with the gap junction protein contribute to cell adhesion, excitability, and electrical coupling [8]. He found that loss of expression of plakophilin-2, an event responsible for an inherited cardiomyopathy, increases the release of ATP in a Cx43-dependent manner. Based on this, he proposed that the connexome could represent a target to interfere with paracrine-mediated initiation of a pro-fibrotic program. Brenda Kwak (Université de Genève, Switzerland) spoke about atherosclerosis, an inflammatory disease of large- and medium-sized arteries and presented data indicating that hemichannels and gap junction channels play a role in atherogenesis [9]. Using mouse models for atherosclerosis, she has shown that Cx37 hemichannels control the initiation of atherosclerosis in mice by inhibiting autocrine ATP-dependent regulation of monocyte adhesion [10]. In addition, arterial blood flow patterns regulate Cx37 expression thereby inducing the formation of distinct communication compartments in the endothelium, with a segregation of athero-prone and -protected regions [11]. Moreover, connexins also play a channel-independent role in atherogenesis since a novel functional interaction between Cx40 and IκBα, a regulatory protein of NF-κB, has been found which could be relevant for the development of atherosclerotic disease.

### Healing wounds

Skin is endowed with an extensive spatially distinct expression of various connexins. Patricia Martin (Glasgow Caledonian University, Scotland) reported changes in connexin expression and function associated with a range of epidermal disorders [12]. At the edge of chronic non-healing wounds Cx43 and Cx26 are up-regulated and blocking channel function or reducing Cx43 expression improves wound closure rates. In hyperproliferative skin disorders, such as psoriasis, Cx26 is dramatically up-regulated, a hallmark for the disease state. She showed that pro-inflammatory challenge by opportunistic skin pathogens prevalent in non-healing wounds and psoriatic plaques induces Cx26 expression in keratinocytes, activates hemichannels and induces expression of pro-inflammatory cytokines [13]. Blocking Cx hemichannel reduces pro-inflammatory mediated events suggesting that specific connexin channel blockers may provide a strategy to reduce skin inflammation.

### Brain diseases

**Fig. 2:**
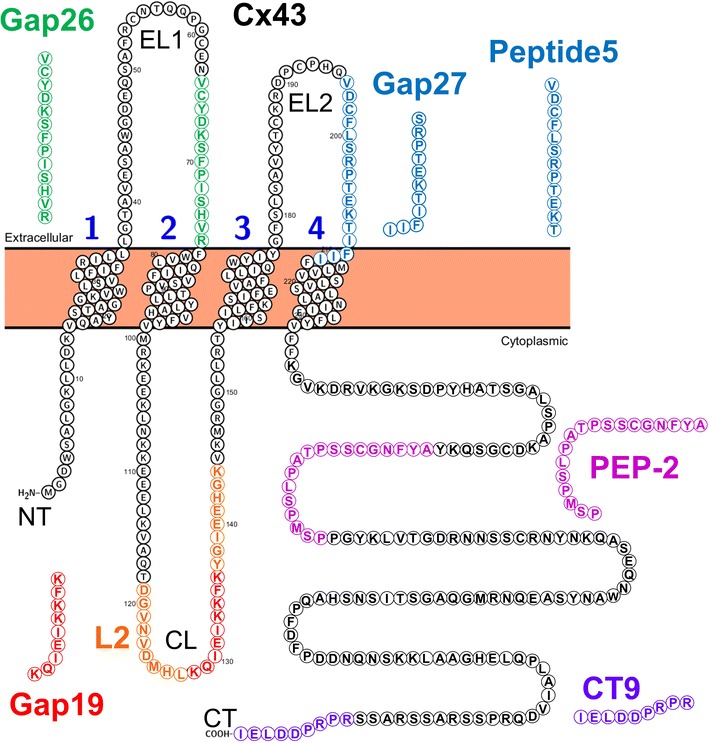
Schmematic of Cx43 amino acid sequence showing portions of the protein which have been targeted with mimetic peptides. See text for details. The commonly used mimetic peptides, Gap26 and Gap 27, are shown for reference. Figure contributed by Dr. L. Leybaert, Ghent University, Belgium

With up to 12 different connexins and 2 pannexins detected in the brain, these channel proteins may also represent therapeutic targets for neurological disorders [14]. Annette Koulakoff (Collège de France, Paris) showed that the expression of Cxs in astroglia is increased at the level of amyloid plaques, a typical histopathological lesion in brains of patients and murine models of Alzheimer’s disease (APP/PS1 mouse). She reported that in this mouse, gap junctional communication (GJC) is not affected in astrocytes, while hemichannels are chronically activated. Cx43 is the main hemichannel contributor activated by the increase in intracellular calcium levels. Importantly, hemichannel activation leads to a release of ATP and glutamate that contributes to maintain a high calcium level placing astrocytes at the center of a vicious cycle. Astroglial targeted Cx43 gene KO in APP/PS1 mice diminishes gliotransmitter release and alleviates neuronal damage, suggesting that blocking hemichannel activity in astrocytes may represent an alternative therapeutic strategy to reduce neuronal suffering in Alzheimer’s disease [15]. Peter Bedner (Bonn University, Germany) analyzed functional properties of astrocytes in hippocampal specimens from patients with mesial temporal lobe epilepsy (MTLE) with and without hippocampal sclerosis (MTLE-HS). He found that the sclerotic human hippocampus is completely devoid of bona fide astrocytes and GJC. Employing a new mouse model of MTLE-HS, he showed that reduction in GJC and the consequential impairment of extracellular potassium clearance precedes neuronal death and the onset of spontaneous seizure activity. A similar feature was observed in another seizure model, hyperthermia-induced febrile convulsions, providing strong evidence that dysfunction of astroglial GJC represents a fundamental mechanism in epileptogenesis [16]. In related studies, Juan Saez (Catholic University of Chile, Santiago) examined neuroinflammation triggered in adult mice treated acutely or chronically with epileptogenic compounds or in the brain of the offspring from mothers treated with high glucocorticoid levels during pregnancy. Under these conditions the activity of pannexin1 and connexin hemichannels is drastically increased in mast cells, microglia and macroglia, including astrocytes. Treatment with a selective connexin hemichannel blocker normalizes the EEG in epilepsy and eliminates convulsions in these mice. The Bedner and Saez results address different functions of these channel proteins, as connexin mediated channels contributing to a functional neuroglial interaction either through gap junction-mediated networking or hemichannel-mediated release/uptake pathways, respectively. Finally, Christian Naus (University of British Columbia, Canada) reported that connexin and pannexin channels provide intercellular pathways through which neuronal, glial, and vascular tissues interact. In the brain, this interaction is highly critical for homeostasis and brain repair after injury. He presented current insights and emerging concepts, particularly the impact of Cx43 and pannexin1, under neuroprotective and neurodegenerative conditions in stroke within the context of astrocytes [17]. Specifically blocking Cx43 hemichannels using the Gap19 mimetic peptide [18] (Fig. 2) provided significant neuroprotection in a mouse ischemic stroke model. Furthermore, he reported that pannexin1 KO mice showed a sex-specific neuroprotective effect in stroke; this could also be shown using the pannexin1 channel blocker, probenecid.

### Cancer

Cancer, and in particular glioma, was also considered. Marc Mesnil (Université de Poitiers, France) noted that the most common brain tumor in adults, glioblastoma (grade IV glioma) is not curable because of recurrence due to high invasion capacity. In a study covering gliomas from grades II to IV (85 tumors), he observed that even if Cx43 expression decreases in higher grade tumors some glioblastoma cells still express it. He mentioned experimental data suggesting that lack of Cx43 expression favors cell proliferation while its presence increases invasion capacity. This was confirmed using human glioblastoma cells in which Cx43 was detected in invadopodia able to digest gelatin. Decreasing its expression by shRNAs demonstrated that Cx43 favors invadopodia growth while its hemichannel function inhibits this process. Preliminary data suggest that Cx43 permits interactions between c-Src and cortactin, necessary for actin polymerization and thus for invadopodia elongation independently from mediation of GJC. Confirmation of these molecular mechanisms might lead to therapeutics targeting Cx43 to prevent invasion and recurrence of glioblastomas [19]. Consistent with these findings, Arantxa Tabernero (University of Salamanca, Spain) noted that c-Src interacts with Cx43 through its SH3 binding domain and phosphorylates Tyr265, providing an SH2 binding domain site with subsequent phosphorylation at Tyr247. As a consequence, GJC is reduced, and Cx43 turnover is initiated. She revealed that the interaction between Cx43 and c-Src inhibits c-Src activity [20]. Also, gain- and loss-of-function experiments, carried out in glioma cells and astrocytes, showed that Cx43 and c-Src are mutually regulated by a phosphorylation/dephosphorylation loop. Accordingly, results obtained with cell-penetrating peptides containing the Src-interacting region show that a short peptidic sequence is sufficient to mimic the effect of Cx43 on the inhibition of c-Src activity. Thus, the region of 266–283 amino acids of Cx43 (PEP-2 in Fig. 2) could help to develop new drugs that target diseases, such as glioma or neuroinflammation, in which the over-activation of c-Src needs to be reduced [21, 22]. Finally, Robert Gourdie (Center for Heart and Regenerative Medicine, Roanoke) indicated that the standard-of-care treatment for glioblastoma involves surgery to remove the tumor, followed by radiation and chemotherapy. Unfortunately, this treatment is largely ineffective as tumors recur and a main reason for this failure is that patients develop resistance to chemotherapy [23, 24]. He mentioned that Cx43 is a key determinant of chemotherapy resistance and that the Cx43 inhibitory peptide αCT1 (CT9 in Fig. 2) recovers the sensitivity of human glioblastoma cells and glioma stem cells to chemotherapy in vitro and in glioblastoma animal models in vivo [25]. To reformulate αCT1 for internal use in brain tumors, αCT1 can be encapsulated in an FDA-approved polymer to provide for its controlled-release in situ following delivery into brain tumors. The release kinetics of αCT1 from the polymer vehicle has been confirmed in vitro over 3 weeks with uptake and dissolution. The controlled-release αCT1 formulation is presently being tested in combination with chemotherapy in a clinical trial in dogs with naturally occurring high-grade glioma. The first of the canine patients entered the protocol in April 2016 and it is expected that 6–8 dogs will have received the combined treatment by the end of 2016. If safety outcomes are favorable, he anticipates seeking regulatory approval from the FDA to advance to clinical trials in humans in 2017.

### Chronic diseases: kidney and lung

Next, the presentation of Christos Chadjichristos (INSERM, Paris) was focused on Cx43 being a new therapeutic target against chronic kidney disease (CKD). Excessive recruitment of monocytes and progression of fibrosis are hallmarks of CKD. Cx43 expression is highly increased within damaged compartments of the kidney during the progression of CKD in humans and rodents. Reduced expression of Cx43 in a hypertension-induced nephropathy model in mice shows a marked decrease of cell adhesion markers leading to reduced monocyte infiltration and interstitial renal fibrosis. Accordingly, functional and histological parameters such as albuminuria and glomerulosclerosis are substantially ameliorated. Furthermore, short-term treatment with Cx43 antisense produced remarkable improvement of renal function and structure in mice at a very advanced stage of hypertension-induced kidney disease [26]. The beneficial effect of Cx43 antisense was observed in additional models of experimental nephropathy confirming that the beneficial effect of Cx43 blockade can be considered as a general protective strategy against renal disease. He concluded that Cx43 might represent a new therapeutic target against the progression of CKD. Marc Chanson (Université de Genève, Switzerland) talked about airway epithelial infection with a focus on connexin and host-pathogen interaction in cystic fibrosis (CF) airway disease. Respiratory tract infections are hallmarks of CF, a hereditary disease caused by mutations of the CF transmembrane conductance regulator (CFTR) gene. An understanding of the interaction between host and pathogen is essential for effective prevention and control of infectious diseases. In this context, he reported that Cx43 in polarized airway epithelial cells is a target of *P. aeruginosa* via toll-like receptor 5 (TLR5) activation, leading to its increased functional expression by means of interplay between p38 and JNK MAPKs [27]. He further showed that GJC modulates airway cell apoptosis, a defense mechanism that is altered after CFTR silencing. He found that CFTR intersects with JNK-signaling to modulate Cx43 expression and apoptosis, suggesting that CFTR dysfunction may alter the apoptosis/inflammation balance in CF.

### Therapeutic strategies

Therapeutic approaches designed to target connexins and pannexins involved in disease were an integral part of this workshop. These consisted of approaches to target specific functional aspects of these channels. Luc Leybaert (Gent University, Belgium) presented connexin hemichannel targeting peptides as tools to protect against cell injury and cell death. He stated that while GJC has major physiological roles, connexin hemichannels are thought to have mostly detrimental effects. As a result, there is a strong need for specific hemichannel blockers that do not affect GJC. He demonstrated that peptides like L2 and Gap19 (Fig. 2) specifically block Cx43-based hemichannels without affecting GJC. Their effect is based on blocking the interaction between the cytoplasmic loop and the C-terminal tail of Cx43. Recent work with the hemichannel blocking peptide RRNYRRNY, the design of which is based on the pharmacophore for CL to CT binding, has the advantage to counteract gap junction channel closure [18, 28]. He also found that this peptide blocks mitochondrial Cx43 hemichannels and mitochondrial calcium ion uptake, thereby protecting against cell death. Finally, he proposed that connexin channels could be targeted at three levels: the gap junction channels, the hemichannels in the plasma membrane and the mitochondrial hemichannels.

As an critical innovative aspect of this meeting, Pharma and Biotech companies, having a common interest in connexins as potential therapeutical targets, revealed their targeting strategies. Gautam Ghatnekar (FirstString Research, USA) gave an overview of their focus on scar prevention, inflammation reduction, wound healing, and complex tissue regeneration. He claimed that Cx43 is a novel therapeutic target with the potential to manipulate the body’s injury response. With this objective FirstString has implemented a 25aa peptide (αCT1) with a compact 2-domain design based on linkage of an antennapedia internalization domain to the C-terminal PDZ binding domain of Cx43 (CT9 in Fig. 2), originally reported by Gourdie and colleagues [29]. When applied on cutaneous wounds, αCT1 restores GJC that is lost in injured tissues and reduces excessive inflammatory responses characterizing chronic wounds [30]. This reestablishes a normal wound repair cascade, accelerates re-epithelialization, and prevents excessive fibrosis. αCT1’s design enables translocation within cells and inhibition of the interaction between Cx43 and zonula occludens-1 (ZO-1). Such inhibition leads to phosphorylation of Ser368 on Cx43, and cellular redistribution of Cx43. The result is a transition of cell-surface Cx43 from hemichannels to gap junction channels, enhancing the stability of gap junction channel aggregates, reducing hemichannel activity and tempering inflammatory responses. FirstString Research is currently advancing a topical αCT1 formulation (Granexin^®^) through pivotal human clinical trials for acute and chronic cutaneous wounds. Three phase 2 (n = 276) clinical trials validate the clinical potential of Granexin^®^ in accelerating the closure of refractory diabetic foot ulcers [31] and venous leg ulcers [32], and in the mitigation of acute surgical incision scars. Results of clinical trials support the tolerability and clinically significant efficacy of αCT1 in the treatment of acute and chronic wounds. Its beneficial effects in cutaneous wound healing translate easily to the treatment of other injury pathologies similarly defined within the context of connexin signaling. Mathieu Charvériat (Theranexus, France) presented data on astroglial connexins and modafinil efficacy. Modafinil is a standard of care used in narcolepsy, a rare disease characterized by excessive daytime sleepiness, and it modulates astroglial GJC [33]. He presented findings in mice on the impact of GJC on the effects of modafinil using flecainide, an anti-arrhythmic registered drug repurposed as an astroglial GJC inhibitor [34]. Indeed, modafinil enhances astrocyte GJC and he demonstrated that this modafinil-induced effect on GJC was prevented by co-administration with flecainide. He also established that flecainide enhanced the wake-promoting and pro-cognitive effects of modafinil; in addition modafinil/flecainide treatment resulted in a decrease in the number of narcoleptic episodes. This study indicates that flecainide improves the pharmacological profile of modafinil, likely through the normalization of GJC in astroglial networks, opening new perspectives in the management of narcolepsy. In fact, Theranexus recently been announced the results of a proof of concept clinical trial in excessive daytime sleepiness (EDS) induced by sleep deprivation in healthy volunteers. THN102 (combination of modafinil and flecainide, repurposed at low dose as a glial connexin modulator) has a beneficial effect on vigilance and cognition throughout the sleep deprivation when compared to the standard of care modafinil. Finally, Rie Hansen and Anneline Nansen (Zealand Pharma A/S, Smedeland, Denmark) spoke about this biotech company’s leading scientific expertise in turning peptides into medicines with several libraries of gap junction modulating compounds. These include a library of 200 Cx43 CT interacting compounds from 40 mer peptides to modified tripeptides, a library of 20 Cx37 CT-interacting peptides, a library related to danegaptide/rotigaptide (zealandpharma.com) that contains 500 compounds, including hexapeptides, cyclic peptides, modified dipeptides and small molecules, a library containing of 150 modified Gap-peptides, GJC/pannexin1 inhibitors against Cx43, 40, 36, 32, 31.1, 26 and pannexin1, and many more. Zealand is willing to share the compounds for scientific investigation under a material transfer agreement. Within the gap junction field Zealand is running a project focused on accelerated dermal wound healing by specific Cx43 inhibition. Furthermore, Zealand has very recently conducted a phase 2 study focused to treat reperfusion injury after acute myocardial infarction, with danegaptide, a modulator of Cx43. Unfortunately, this study did not meet its primary endpoint. Another company, CoDa Therapeutics Inc., was, unfortunately, unable to participate in the meeting. CoDa has three connexin channel modulators in clinical development, in the first instance for chronic skin wounds and ocular disease. These are an antisense oligodeoxynucleotide (Nexagon^®^) which transiently downregulates Cx43 protein expression, an extracellular acting Cx43 peptidomimetic that can be delivered locally or systemically (Peptagon™) (Peptide 5 in Fig. 2) and a small molecule for systemic or oral delivery (HCB1019) [35]. The latter two are specifically used to target hemichannels. Nexagon^®^ and HCB1019 are Phase III ready. CoDa has focused significant activity on the connexin hemichannel and has demonstrated efficacy in a number of skin, eye and central nervous system injury and disease animal models where hemichannel block, or transient down regulation of Cx43, reduces oedema, inflammation, vascular leak and lesion spread [36]. In human eye burns for example, reduced inflammation and recovery of vascular integrity following down regulation of Cx43, leads to epithelial recovery and healing [37].

## Summary and future directions

Considerable insights continue to emerge regarding the biology of connexins and pannexins, and their possible roles in health and disease. With the knowledge of tissue distribution combined with deeper analysis of structure and the functions of these channel proteins, novel therapeutic approaches are impacting various diseases. Presentations and discussions held during this workshop revealed new lines of research and identified overlapping fields of interest between fundamental life science and biotech initiatives. These can be grouped into several categories. (1) With regard to channel functions, drugs specifically targeting connexin channel versus hemichannel configurations will prove beneficial. These have focused on small peptide candidates. In this regard, Gap19 blocks Cx43 hemichannels while αCT1 enhances Cx43 gap junction plaque formation. Peptides containing the Src-interacting region can modulate kinase interactions with Cx43. Considerable efforts continue to be focused on exploring novel peptide therapeutics. (2) Non-channel functions of Cx43 are also being identified and their targeting should also open the way to new intervention strategies. (3) Specific effects can also be expected by targeting the “connexome” of interacting proteins and their associated signaling pathways. (4) Finally, combinatorial approaches using connexin targeting combined with other drugs are emerging as a promising strategy. Based on the outcome of this workshop, there is considerable optimism with regard to bridging the gap to therapeutic strategies based on connexin/pannexin biology.

## Abbreviations

APP/PSI: amyloid precursor protein/presenelin-1
CF: cystic fibrosis
CFTR: CF transmembrane conductance regulator
CKD: chronic kidney disease
Cx43: connexin43
Cx37: connexin37
Cx40: connexin40
Cx26: connexin26
GJC: gap junction coupling
IκBα: inhibitor of kappa B
JNK MAPK: jun N-terminal kinase
KO: knockout
MTLE: mesial temporal lobe epilepsy
MTLE-HS: mesial temporal lobe epilepsy with hippocampal sclerosis
NF-κB: nuclear factor kappa B
ODDD: oculodentodigital dysplasia
P38 MAPK: P38 mitogen-activated protein kinases
TLR5: toll-like receptor 5
ZO-1: zonula occludens-1

## References

[1] Laird DW, Lampe PD, Johnson RG (2015). Cellular small talk. Sci Am.

[2] Vinken M (2015). Connexin hemichannels: novel mediators of toxicity. Arch Toxicol.

[3] Kelly JJ, Simek J, Laird DW (2015). Mechanisms linking connexin mutations to human diseases. Cell Tissue Res.

[4] Bedner P, Steinhauser C, Theis M (2012). Functional redundancy and compensation among members of gap junction protein families?. Biochim Biophys Acta.

[5] Penuela S, Harland L, Simek J, Laird DW (2014). Pannexin channels and their links to human disease. Biochem J.

[6] Solan JL, Lampe PD (2016). Kinase programs spatiotemporally regulate gap junction assembly and disassembly: effects on wound repair. Semin Cell Dev Biol.

[7] Leo-Macias A, Agullo-Pascual E, Delmar M (2016). The cardiac connexome: non-canonical functions of connexin43 and their role in cardiac arrhythmias. Semin Cell Dev Biol.

[8] Herve JC, Derangeon M, Sarrouilhe D, Giepmans BN, Bourmeyster N (2012). Gap junctional channels are parts of multiprotein complexes. Biochim Biophys Acta.

[9] Kwak BR, Back M, Bochaton-Piallat ML, Caligiuri G, Daemen MJ, Davies PF, Hoefer IE, Holvoet P, Jo H, Krams R (2014). Biomechanical factors in atherosclerosis: mechanisms and clinical implications. Eur Heart J.

[10] Wong CW, Christen T, Roth I, Chadjichristos CE, Derouette JP, Foglia BF, Chanson M, Goodenough DA, Kwak BR (2006). Connexin37 protects against atherosclerosis by regulating monocyte adhesion. Nat Med.

[11] Pfenniger A, Wong C, Sutter E, Cuhlmann S, Dunoyer-Geindre S, Mach F, Horrevoets AJ, Evans PC, Krams R, Kwak BR (2012). Shear stress modulates the expression of the atheroprotective protein Cx37 in endothelial cells. J Mol Cell Cardiol.

[12] Martin PE, van Steensel M (2015). Connexins and skin disease: insights into the role of beta connexins in skin homeostasis. Cell Tissue Res.

[13] Donnelly S, English G, de Zwart-Storm EA, Lang S, van Steensel MA, Martin PE (2012). Differential susceptibility of Cx26 mutations associated with epidermal dysplasias to peptidoglycan derived from *Staphylococcus aureus* and *Staphylococcus epidermidis*. Exp Dermatol.

[14] Giaume C, Leybaert L, Naus CC, Saez JC (2013). Connexin and pannexin hemichannels in brain glial cells: properties, pharmacology, and roles. Front Pharmacol.

[15] Yi C, Mei X, Ezan P, Mato S, Matias I, Giaume C, Koulakoff A (2016). Astroglial connexin43 contributes to neuronal suffering in a mouse model of Alzheimer’s disease. Cell Death Dis..

[16] Bedner P, Dupper A, Huttmann K, Muller J, Herde MK, Dublin P, Deshpande T, Schramm J, Haussler U, Haas CA (2015). Astrocyte uncoupling as a cause of human temporal lobe epilepsy. Brain.

[17] Freitas-Andrade M, Naus CC (2016). Astrocytes in neuroprotection and neurodegeneration: the role of connexin43 and pannexin1. Neuroscience.

[18] Abudara V, Bechberger J, Freitas-Andrade M, De Bock M, Wang N, Bultynck G, Naus CC, Leybaert L, Giaume C (2014). The connexin43 mimetic peptide Gap19 inhibits hemichannels without altering gap junctional communication in astrocytes. Front Cell Neurosci.

[19] Sin WC, Crespin S, Mesnil M (2012). Opposing roles of connexin43 in glioma progression. Biochim Biophys Acta.

[20] Herrero-Gonzalez S, Gangoso E, Giaume C, Naus CC, Medina JM, Tabernero A (2010). Connexin43 inhibits the oncogenic activity of c-Src in C6 glioma cells. Oncogene.

[21] Gangoso E, Thirant C, Chneiweiss H, Medina JM, Tabernero A (2014). A cell-penetrating peptide based on the interaction between c-Src and connexin43 reverses glioma stem cell phenotype. Cell Death Dis.

[22] González-Sánchez A, Jaraíz-Rodríguez M, Domínguez-Prieto M, Herrero-González S, Medina JM, Tabernero A (2016). Connexin43 recruits PTEN and CSK to inhibit c-Src in glioma cells and astrocytes. Oncotarget..

[23] Gielen PR, Aftab Q, Ma N, Chen VC, Hong X, Lozinsky S, Naus CC, Sin WC (2013). Connexin43 confers temozolomide resistance in human glioma cells by modulating the mitochondrial apoptosis pathway. Neuropharmacology.

[24] Murphy SF, Varghese RT, Lamouille S, Guo S, Pridham KJ, Kanabur P, Osimani AM, Sharma S, Jourdan J, Rodgers CM (2016). Connexin 43 inhibition sensitizes chemoresistant glioblastoma cells to temozolomide. Cancer Res.

[25] Yusubalieva GM, Baklaushev VP, Gurina OI, Zorkina YA, Gubskii IL, Kobyakov GL, Golanov AV, Goryainov SA, Gorlachev GE, Konovalov AN (2014). Treatment of poorly differentiated glioma using a combination of monoclonal antibodies to extracellular connexin-43 fragment, temozolomide, and radiotherapy. Bull Exp Biol Med.

[26] Abed A, Toubas J, Kavvadas P, Authier F, Cathelin D, Alfieri C, Boffa JJ, Dussaule JC, Chatziantoniou C, Chadjichristos CE (2014). Targeting connexin 43 protects against the progression of experimental chronic kidney disease in mice. Kidney Int.

[27] Losa D, Kohler T, Bellec J, Dudez T, Crespin S, Bacchetta M, Boulanger P, Hong SS, Morel S, Nguyen TH (2014). Pseudomonas aeruginosa-induced apoptosis in airway epithelial cells is mediated by gap junctional communication in a JNK-dependent manner. J Immunol.

[28] Wang N, De Vuyst E, Ponsaerts R, Boengler K, Palacios-Prado N, Wauman J, Lai CP, De Bock M, Decrock E, Bol M (2013). Selective inhibition of Cx43 hemichannels by Gap19 and its impact on myocardial ischemia/reperfusion injury. Basic Res Cardiol.

[29] Hunter AW, Barker RJ, Zhu C, Gourdie RG (2005). Zonula occludens-1 alters connexin43 gap junction size and organization by influencing channel accretion. Mol Biol Cell.

[30] Ghatnekar GS, O’Quinn MP, Jourdan LJ, Gurjarpadhye AA, Draughn RL, Gourdie RG (2009). Connexin43 carboxyl-terminal peptides reduce scar progenitor and promote regenerative healing following skin wounding. Regen Med.

[31] Grek CL, Prasad GM, Viswanathan V, Armstrong DG, Gourdie RG, Ghatnekar GS (2015). Topical administration of a connexin43-based peptide augments healing of chronic neuropathic diabetic foot ulcers: a multicenter, randomized trial. Wound Repair Regen.

[32] Ghatnekar GS, Grek CL, Armstrong DG, Desai SC, Gourdie RG (2015). The effect of a connexin43-based peptide on the healing of chronic venous leg ulcers: a multicenter, randomized trial. J Invest Dermatol.

[33] Liu X, Petit JM, Ezan P, Gyger J, Magistretti P, Giaume C (2013). The psychostimulant modafinil enhances gap junctional communication in cortical astrocytes. Neuropharmacology.

[34] Duchene A, Perier M, Zhao Y, Liu X, Thomasson J, Chauveau F, Pierard C, Lagarde D, Picoli C, Jeanson T (2016). Impact of astroglial connexins on modafinil pharmacological properties. Sleep.

[35] Becker DL, Phillips AR, Duft BJ, Kim Y, Green CR (2016). Translating connexin biology into therapeutics. Semin Cell Dev Biol.

[36] Danesh-Meyer HV, Zhang J, Acosta ML, Rupenthal ID, Green CR (2016). Connexin43 in retinal injury and disease. Prog Retin Eye Res.

[37] Ormonde S, Chou CY, Goold L, Petsoglou C, Al-Taie R, Sherwin T, McGhee CN, Green CR (2012). Regulation of connexin43 gap junction protein triggers vascular recovery and healing in human ocular persistent epithelial defect wounds. J Membr Biol.

